# Successful Treatment of Advanced Intrahepatic Cholangiocarcinoma With a High Tumor Mutational Burden and PD-L1 Expression by PD-1 Blockade Combined With Tyrosine Kinase Inhibitors: A Case Report

**DOI:** 10.3389/fimmu.2021.744571

**Published:** 2021-09-17

**Authors:** Ze Zhang, Wenwen Zhang, Hongguang Wang, Bingyang Hu, Zhanbo Wang, Shichun Lu

**Affiliations:** ^1^Medical School of Chinese People’s Liberation Army (PLA), Beijing, China; ^2^Faculty of Hepato-Pancreato-Biliary Surgery, Chinese PLA General Hospital, Beijing, China; ^3^Institute of Hepatobiliary Surgery of Chinese PLA, Beijing, China; ^4^Key Laboratory of Digital Hepatobiliary Surgery, PLA, Beijing, China; ^5^Department of Hepatobiliary Surgery, Cancer Hospital Chinese Academy of Medical Sciences, Beijing, China; ^6^Department of Pathology, Chinese PLA General Hospital, Beijing, China

**Keywords:** intrahepatic cholangiocarcinoma, immunotherapy, conversion therapy, PD-L1, TMB

## Abstract

Advanced intrahepatic cholangiocarcinoma (iCCA) is not suitable for surgical treatment. Guided by the concept of precision medicine, preoperative systematic treatment may reshape the clinical outcomes of advanced intrahepatic cholangiocarcinoma patients. We describe the case of a 38-year-old female who has been diagnosed with stage IV intrahepatic cholangiocarcinoma with a high tumor mutational burden and positively programmed death-ligand 1 (PD-L1) expression. The patient was treated with programmed cell death 1 (PD-1) inhibitors combined with tyrosine kinase inhibitors (TKIs). After 7 cycles of combination therapy, she underwent radical resection and no tumor cells were found in the postoperative histopathological examination. In addition, the patient’s survival time had reached 25 months, as of August 2021. To date, this is the first case of successful radical resection after combined immunotherapy with TKIs for advanced PD-L1-positive intrahepatic cholangiocarcinoma with a high tumor mutational burden (TMB). The case provides a new approach to the treatment of advanced intrahepatic cholangiocarcinoma.

## Introduction

Intrahepatic cholangiocarcinoma is the second most common primary liver cancer after hepatocellular carcinoma, and the incidence rate of iCCA has been on the rise over recent decades (1). In the early stages of iCCA, curative resection is the preferred treatment option, but a majority of patients (approximately 60-70%) are diagnosed with advanced-stage disease that is not suitable for radical resection with curative intent (2). For patients with unresectable iCCA, the current standard of systemic treatment is gemcitabine combined with platinum-based compounds, but the median survival is less than 12 months (3). Systemic and local therapy prior to surgery may increase the proportion of patients who are eligible for radical resection and reduce the postoperative recurrence rate. The traditional conversion therapies for advanced iCCA include systemic chemotherapy, transarterial chemoembolization (TACE), transarterial selective internal radiation/radioembolization therapy, and hepatic artery infusion (4). However, the optimal conversion treatment strategy and related survival benefits remain unclear.

In recent years, immunotherapy has yielded encouraging results in a variety of cancers, and National Comprehensive Cancer Network (NCCN) guidelines recommend pembrolizumab as a treatment choice for advanced cholangiocarcinoma with deficient mismatch repair (dMMR) and high microsatellite instability (MSI-H) (5). The relationship between PD-L1 expression in tumors and the efficacy of PD-1 inhibitors are still being investigated (6–8). PD-L1 is expressed in tumors from patients with iCCA, suggesting the feasibility of targeting the PD-1/PD-L1 pathway (9). For example, a study has preliminarily demonstrated that the expression of PD-L1 in tumor tissues of iCCA patients can be used as a biomarker to predict the efficacy of PD-1 inhibitor therapy (10). Furthermore, preliminary studies of immunotherapy combined with targeted therapy for iCCA are ongoing and have shown promising therapeutic prospects (11). Nevertheless, surgery for advanced iCCA following a combination of immunotherapy combined with targeted therapy has not been reported to date.

**Figure 1:**
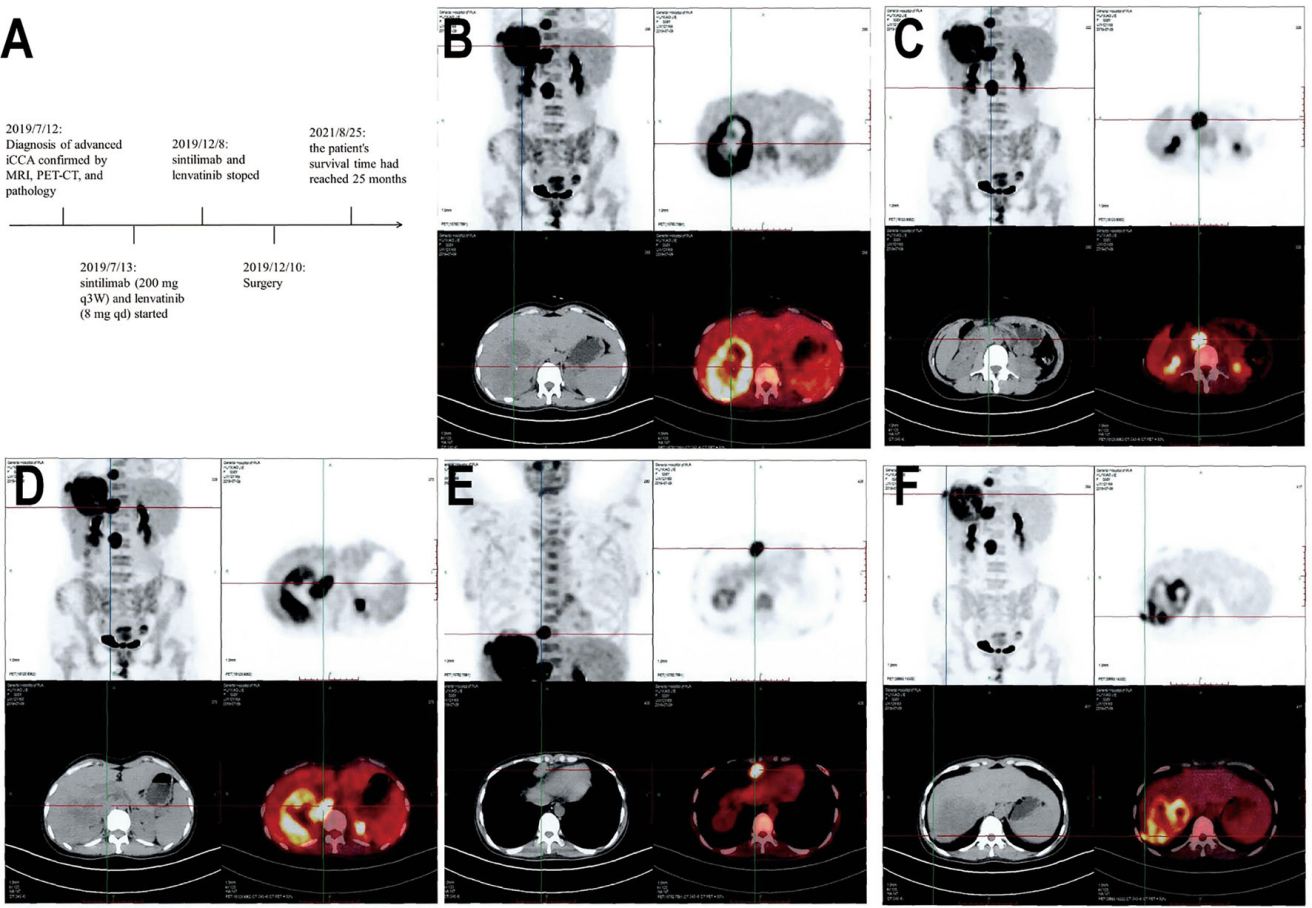
Timeline and PET-CT examination. **(A)** Indicates initial diagnosis, medication treatment, surgery, and follow-up timeline of the patient. **(B)** Shows a large mass in the right lobe of the liver with increased radioactive uptake of the solid component. **(C)** Shows multiple enlarged lymph nodes adjacent to the abdominal aorta with increased radioactive uptake. **(D)** Shows increased nodular radioactive uptake in the inferior vena cava. **(E, F)** Show anterior costal diaphragm nodule and right diaphragmatic nodule with increased radioactive uptake.

In the present report, we performed the first case study investigating a patient suffering from advanced intrahepatic cholangiocarcinoma with a high TMB and high expression of PD-L1 feature. Guided by the concept of precision medicine, the patient underwent an operation after successful conversion therapy with PD-1 blockade and lenvatinib. Encouragingly, this patient achieved pathological complete response (pCR) and long-term survival. **Figure 1A** shows the diagnosis, treatment and follow-up timeline for this patient.

## Case Presentation

**Figure 2:**
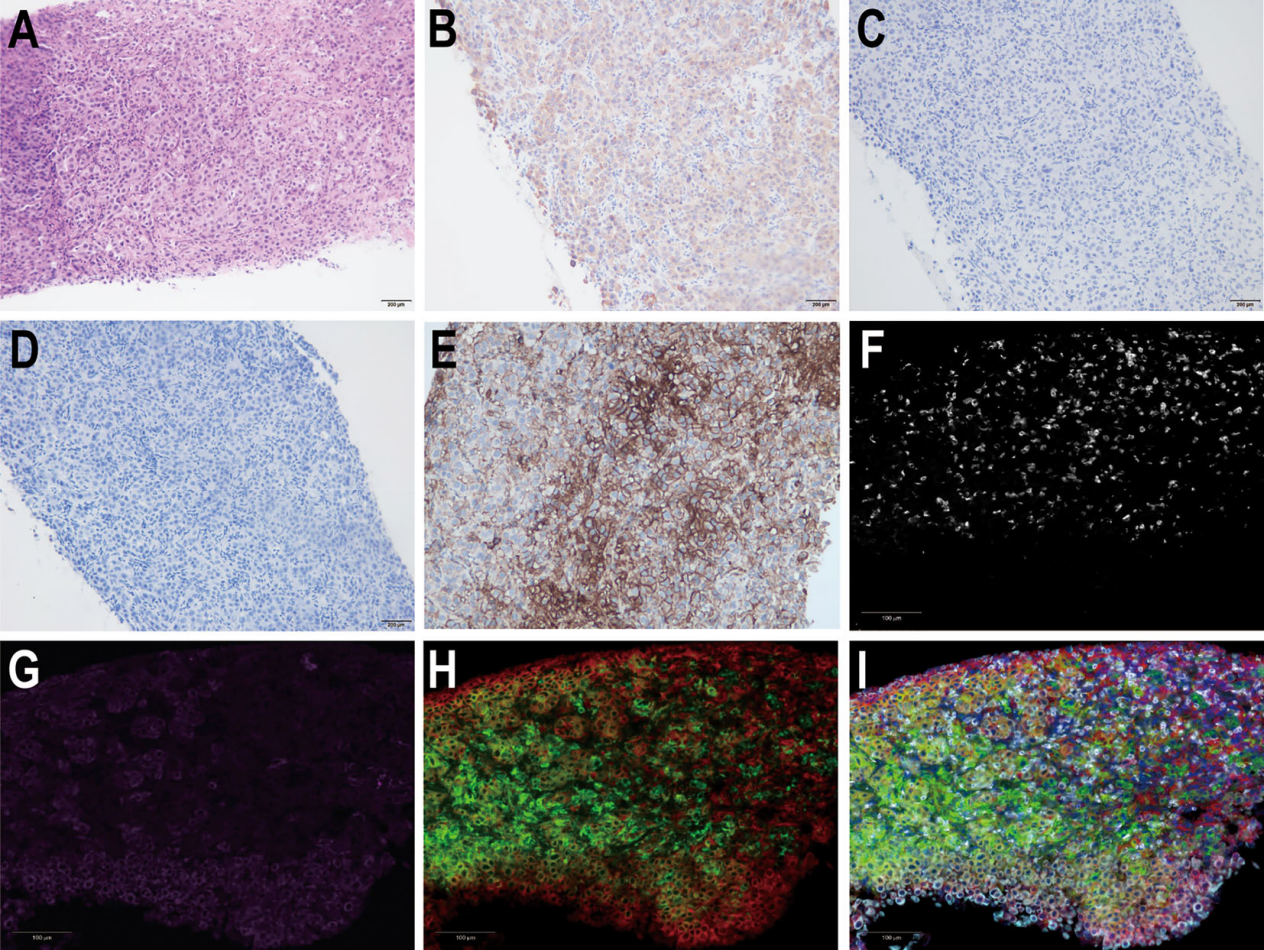
Hematoxylin-eosin (HE) staining and immunohistochemical analysis of liver tumor tissue from the needle biopsy. Pathological images show **(A)** HE staining (200×), **(B)** positive CK19 staining, **(C)** negative AFP staining, and **(D)** negative hepatocyte staining. **(E)** Immunohistochemical staining for PD-L1 expression (200×). Multiple fluorescence immunohistochemical images show **(F)** the CD8+T cells, **(G)** the natural killer (NK) cells, **(H)** the macrophages, and **(I)** the merged images of the previous three images.

**Figure 3:**
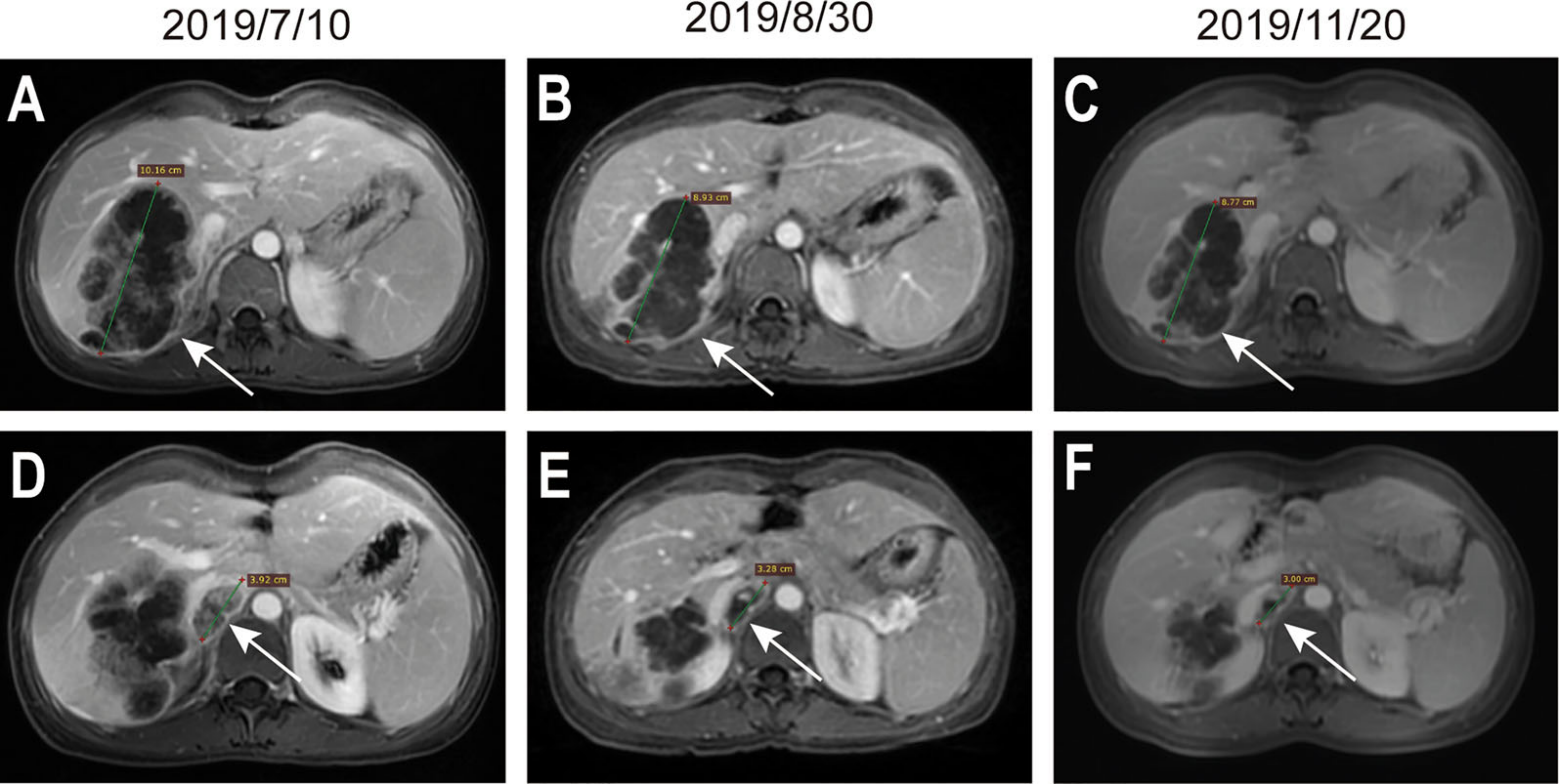
MRI evaluation during preoperative systemic treatment. **(A–C)** indicate the maximum diameter of the lesion located in the liver, the maximum diameter of the lesion in **(A–C)** were 10.16cm, 8.93cm, and 8.77cm. **(D–F)** indicate the lesion located in the tumorigenic thrombus of the inferior vena cava, the maximum diameter of the lesion in **(D–F)** were 3.92cm, 3.28cm, and 3.00cm. Arrows in the figures indicate the position of lesions.

A 38-year-old female was admitted to the hospital on July 6, 2019, due to recurrent right upper abdominal pain. She had a history of chronic hepatitis B infection for 7 years that had not been treated. The patient’s performance status score was 1. Alpha-fetoprotein (AFP), abnormal prothrombin, carcinoembryonic antigen (CEA) and cancer antigen 19-9 (CA19-9) levels were within the normal range. Upper abdominal magnetic resonance imaging (MRI) revealed a mass in the right hepatic lobe with involvement of the inferior vena cava, along with retroperitoneal and right cardio-phrenic angle lymphatic metastases. Positron emission tomography-computed tomography (PET-CT) scans showed a huge mass with uneven increased metabolism in the right lobe of the liver, increased metabolism of the inferior vena cava nodule, increased metabolism of the right diaphragmatic nodule, and hypermetabolic lymph nodes near the abdominal aorta and anterior costal diaphragm (**Figures 1B–F**). The patient underwent a tumor needle biopsy guided by ultrasound on July 12, 2019. The pathohistological analysis of the tumor revealed poorly differentiated adenocarcinoma with necrotic components (**Figure 2A**). Upon immunohistochemical (IHC) analysis, the tumor was found to be positive for cytokeratin 18 and cytokeratin 19 (**Figure 2B**) while being negative for AFP and hepatocyte (**Figures 2C, D**), predisposed to cholangiocarcinoma. All indications suggested a diagnosis of stage IV iCCA (T2N1M1) according to the American Joint Committee on Cancer (AJCC) Cancer Staging Handbook,8th edition (12). With the patient’s consent, whole-exome sequencing (WES) and deep sequencing of a panel of 733 genes was performed with the tissue obtained by the needle biopsy. The tissue was also analyzed for the expression of PD-L1 and the abundance of tumor-infiltrating lymphocytes (TILs). The tumor mutational burden was determined to be 21.77 mutations/Mb and defined as TMB-high (13). A total of 74 neoantigens were detected. No SNVs were detected in PMS2, MSH2, MSH6, or MLH1, suggesting pMMR, and the MSI status was stable. Prominent lymphocytes infiltration were observed in both tumor and stromal region, primarily CD8+ T cell, natural killer (NK) cells, and macrophages (**Figures 2F–I**). The density of these infiltrated lymphocytes at the tumor core were 1594 cells/mm^2^, 986 cells/mm^2^, and 3980 cells/mm^2^ respectively. The tumor proportion score (TPS) of the PD-L1 expression level was 70% according to Allred criteria using the 22C3 monoclonal mouse anti-human PD-L1 antibody (**Figure 2E**). Based on these results, combined immunotherapy with multi-kinase inhibitor therapy was administered. After treatment with sintilimab (200 mg q3W) and lenvatinib (8 mg qd) for 7 cycles (21 weeks), MRI showed that the lesions in the right hepatic lobe and inferior vena cava had significantly decreased in size (**Figure 3**). The patient’s clinical efficacy can be assessed as stable disease (SD) according to the Response Evaluation Criteria in Solid Tumors (version 1.1) (14). The patient experienced only grade 1 adverse events of nausea and vomiting according to the standard CTCAE5.0 criteria.

**Figure 4:**
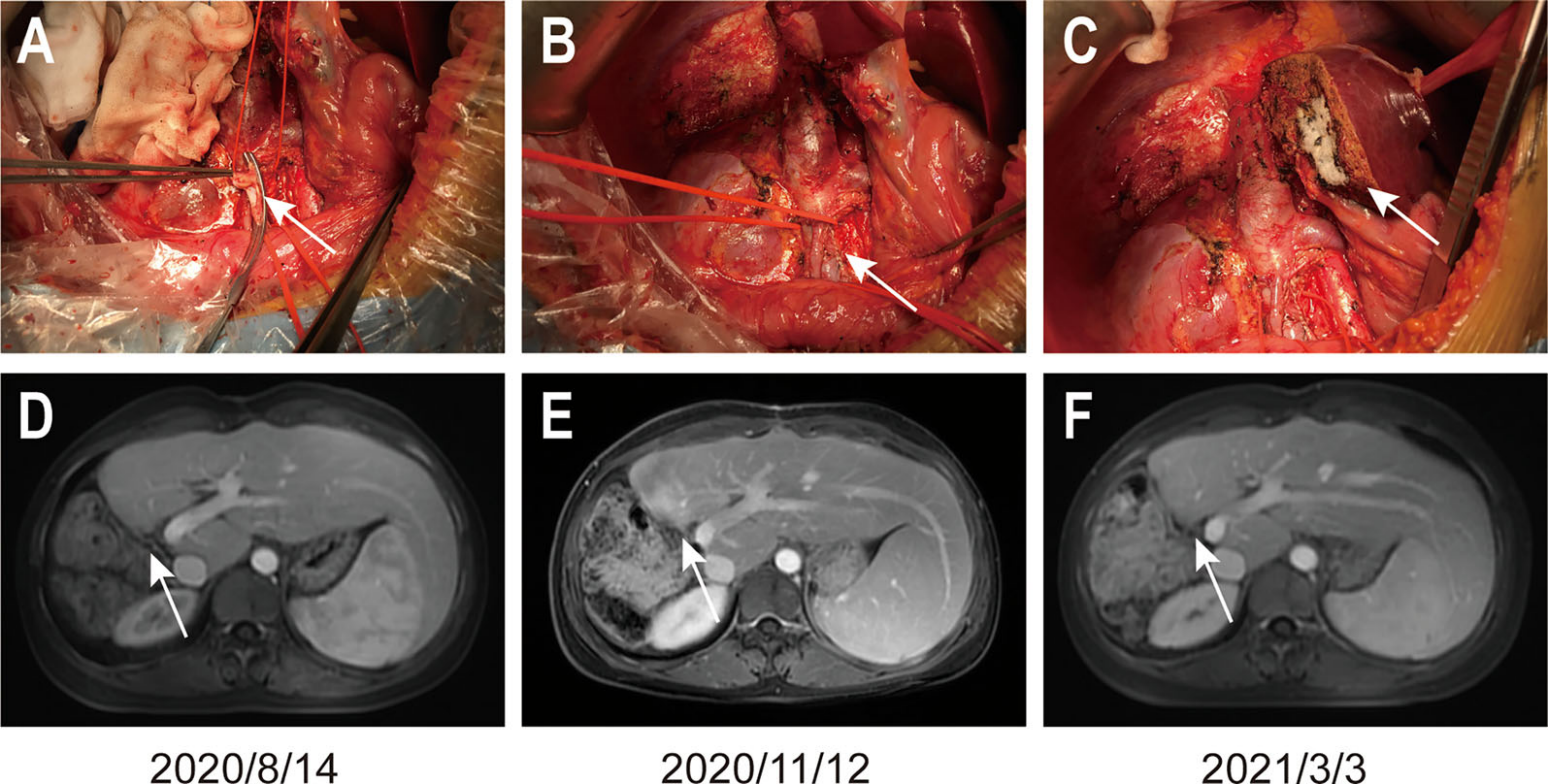
Images of the operation and the MRI reexamination after surgery. **(A)** shows the inferior vena cava embolectomy, **(B)** shows the sutured inferior vena cava, and **(C)** shows the liver incisal margin. Arrows in **(A, B)** figures indicate the position described above. **(D–F)** show the MRI reexamination after surgery. Arrows in **(D–F)** figures indicate the liver resection margin.

The patient underwent right hemihepatectomy, retroperitoneal lymphadenectomy, right cardio-phrenic angle lymphadenectomy, and inferior vena cava embolectomy on December 10, 2019 (**Figures 4A–C**). The patient recovered well after surgery and no serious postoperative complications occurred. The total size of the lesion area in the postoperative specimen was 8.5x6x6cm. No tumor was found in the liver margin, the liver lesion area, groups 8a, 13, 16a, and 16b, the right cardio-phrenic angle lymphatic tissue, or the tumorigenic thrombus of inferior vena cava after surgery. Extensive infiltration of T cells were observed in tumor margin and stromal. The patient underwent periodical MRI reexamination after surgery, which showed no recurrence in the coelom until March 3, 2021 (**Figures 4D–F**).

## Discussion

This case showed that negative surgical margins (R0 resection) can be achieved in a patient with advanced iCCA who received conversion therapy. Furthermore, the result suggests that the combination of lenvatinib with PD-1 inhibitors is an effective and safe conversion therapy strategy.

For primary liver cancer, the downstaging or conversion therapy approaches have been reported over 20 years ago (15, 16), but due to the lack of powerful systemic drugs at that time, the previous conversion therapy strategies mainly focused on local treatment or chemotherapy (17–20). Besides, there is a lack of research on conversion therapy for advanced iCCA currently (21).

Several clinical trials on immunotherapy of advanced biliary tract cancers are ongoing, some preliminary results were favorable (11, 22–24). A clinical trial of the combination of lenvatinib with immune checkpoint inhibitors resulted in an overall response rate (ORR) of 21.4% and disease control rate (DCR) of 92.9% in 14 patients with advanced iCCA who had received more than 2 prior anticancer therapy regimens (10). Several cases had also revealed supporting evidence on the promising effect of combined immunotherapy for advanced iCCA patients. For example, advanced iCCA patients with high TMB, high rate of insertion-deletion mutations (INDELS), and/or positive PD-L1 expression benefited from immunotherapy combined with chemotherapy (25, 26). These preliminary clinical trial results indicated that advanced iCCA patients with high TMB and positive PD-L1 expression were strongly associated with better immunotherapy or its combination with targeted or chemotherapy response (11).

Under the guidance of next-generation sequencing and tumor immune microenvironment testing, our case report suggests that immunotherapy combined with targeted therapy have more than a lethal effect on intrahepatic lesions but also can act on metastatic lesions in distant lymph node and tumorigenic thrombus, which may provide a superior conversion therapy option for unresectable iCCA patients and largely improve their benefit. But to clarify the accurate efficacy and the survival time benefit of this combination strategy, a prospective, multicenter, randomized controlled clinical study is necessary.

## Conclusions

Our case provides a new insight that PD-1 blockade combined with TKIs can successfully convert advanced PD-L1-positive iCCA with a high TMB into resectable iCCA, and no tumor cells were found in the postoperative histopathological examination. The patient’s survival time had reached 25 months, as of August 2021.

## Data Availability Statement

The original contributions presented in the study are included in the article’s **Supplementary Material**. Further inquiries can be directed to the corresponding author.

## Ethics Statement

The studies involving human participants were reviewed and approved by the ethic committee of the Chinese PLA General Hospital (Approval No. S2018-111-01). The patients/participants provided their written informed consent to participate in this study. Written informed consent was obtained from the individual(s) for the publication of any potentially identifiable images or data included in this article.

## Author Contributions

ZZ and WZ were responsible for WES, the whole-exome data analysis, the collection and analysis of clinical data. ZW supervised the pathology interpretation of the patient tissue samples. SL, HW, and BH participated in the operation, took care of patients, and communicated with patients. ZZ was responsible for writing the manuscript. SL was responsible for the analysis of data, data interpretation, and revision. All authors contributed to the article and approved the submitted version.

## Funding

This study was funded by the National Key R&D Program of China (2017YFA0103003 and 2017YFA0103000), and the National Natural Science Foundation of China (81670950).

## Conflict of Interest

The authors declare that the research was conducted in the absence of any commercial or financial relationships that could be construed as a potential conflict of interest.

## Publisher’s Note

All claims expressed in this article are solely those of the authors and do not necessarily represent those of their affiliated organizations, or those of the publisher, the editors and the reviewers. Any product that may be evaluated in this article, or claim that may be made by its manufacturer, is not guaranteed or endorsed by the publisher.

## Abbreviations

iCCA, intrahepatic cholangiocarcinoma; PD-L1, programmed death-ligand 1; PD-1, programmed cell death 1; TKIs, tyrosine kinase inhibitors; AFP, Alpha-fetoprotein; TMB, tumor mutational burden; MRI, magnetic resonance imaging; CTCAE5.0, Common Terminology Criteria for Adverse Events version 5.0.
